# Low Intensity Pulsed Ultrasound Enhanced Mesenchymal Stem Cell Recruitment through Stromal Derived Factor-1 Signaling in Fracture Healing

**DOI:** 10.1371/journal.pone.0106722

**Published:** 2014-09-02

**Authors:** Fang-Yuan Wei, Kwok-Sui Leung, Gang Li, Jianghui Qin, Simon Kwoon-Ho Chow, Shuo Huang, Ming-Hui Sun, Ling Qin, Wing-Hoi Cheung

**Affiliations:** 1 Department of Orthopaedics and Traumatology, Clinical Sciences Building, The Chinese University of Hong Kong, Shatin, New Territories, Hong Kong SAR, China; 2 Translational Medicine Research & Development Center, Institute of Biomedical and Health Engineering, Shenzhen Institutes of Advanced Technology, Chinese Academy of Sciences, Shenzhen, China; Rush University Medical Center, United States of America

## Abstract

Low intensity pulsed ultrasound (LIPUS) has been proven effective in promoting fracture healing but the underlying mechanisms are not fully depicted. We examined the effect of LIPUS on the recruitment of mesenchymal stem cells (MSCs) and the pivotal role of stromal cell-derived factor-1/C-X-C chemokine receptor type 4 (SDF-1/CXCR4) pathway in response to LIPUS stimulation, which are essential factors in bone fracture healing. For *in vitro* study, isolated rat MSCs were divided into control or LIPUS group. LIPUS treatment was given 20 minutes/day at 37°C for 3 days. Control group received sham LIPUS treatment. After treatment, intracellular CXCR4 mRNA, SDF-1 mRNA and secreted SDF-1 protein levels were quantified, and MSCs migration was evaluated with or without blocking SDF-1/CXCR4 pathway by AMD3100. For *in vivo* study, fractured 8-week-old young rats received intracardiac administration of MSCs were assigned to LIPUS treatment, LIPUS+AMD3100 treatment or vehicle control group. The migration of transplanted MSC to the fracture site was investigated by *ex vivo* fluorescent imaging. SDF-1 protein levels at fracture site and in serum were examined. Fracture healing parameters, including callus morphology, micro-architecture of the callus and biomechanical properties of the healing bone were investigated. The *in vitro* results showed that LIPUS upregulated SDF-1 and CXCR4 expressions in MSCs, and elevated SDF-1 protein level in the conditioned medium. MSCs migration was promoted by LIPUS and partially inhibited by AMD3100. *In vivo* study demonstrated that LIPUS promoted MSCs migration to the fracture site, which was associated with an increase of local and serum SDF-1 level, the changes in callus formation, and the improvement of callus microarchitecture and mechanical properties; whereas the blockade of SDF-1/CXCR4 signaling attenuated the LIPUS effects on the fractured bones. These results suggested SDF-1 mediated MSCs migration might be one of the crucial mechanisms through which LIPUS exerted influence on fracture healing.

## Introduction

Millions of fractures occur annually as a result of traumatic injuries or pathological conditions. Although most fractures will successfully heal within a few months, a considerable proportion of fracture cases still result in delayed healing [1], which may prolong treatment period and increase morbidity of the patients.

Mesenchymal stem cells (MSCs) are multipotent stromal cells able to differentiate into many cell types and contribute to the regeneration of musculoskeletal tissues such as bone, cartilage, tendon, adipose, and muscle [2]–[4]. It is widely accepted that MSCs are normally retained in the special niches of different adult tissues. In stressful situations such as injury, when there is a need for tissue repair and to maintain tissue homeostasis, MSCs can be recruited to the site of injury and contribute to the repair process. When bone integrity is disrupted after fracture, the bone tissue would enter a healing process that is generally divided into three overlapping phases including the inflammation, soft and hard callus formation, and the callus remodeling [5]. The damage of blood vessel and other tissues lead to local tissue bleeding and hypoxia. This process will trigger the inflammatory cascade [6]. In this early inflammatory phase of fracture healing, many types of cytokines, such as interleukin 6 (IL-6) and stromal cell-derived factor-1 (SDF-1), released from the damaged bone facilitate the egress of MSCs from the periosteum and bone marrow into the blood stream, which rapidly accumulate and engraft at fracture site, and initiate bone regeneration process [3], [7], [8]. Although the interactions between cytokines and MSCs in bone repair remain controversial, many studies found that MSCs expressed both SDF-1 and CXCR4 genes [9]–[11], and SDF-1/CXCR4 signaling is critical for the recruitment of MSCs to the fracture site during fracture healing. Granero-Molto *et al.* found that implanted MSCs were recruited to the fracture site in an exclusively CXCR4-dependent manner [12]. Kitaori *et al.* showed that SDF-1 level was elevated in the periosteum of injured bone, which recruited MSCs homing to the graft bone at the fracture site and promoted endochondral bone formation [8].

Low intensity pulsed ultrasound (LIPUS) has been reported to be effective in promoting fracture healing in both animal models and clinical trials [13]–[16]. In brief, the beneficial effects of LIPUS on fracture healing include the decrease in healing time at the tissue level, and the increase in the cellular responses including osteogensis-related gene expression [17], protein synthesis and cell proliferation [18]. The mechanical stimulation produced by the pressure waves of LIPUS on bone can result in series of biochemical events at cellular level [19], [20]. However, the detailed mechanism through which LIPUS stimulates tissues remains unclear. Although osteocytes have been considered as the primary mechanosensors in bone, convincing data show that MSCs also have the ability to sense and respond to physical stimuli [21]–[23]. To date, very little is known about how physical stimuli affect MSCs mobilization. One possible mechanism through which LIPUS enhances fracture healing is through the enhancement in MSC recruitment. A recent report has demonstrated that LIPUS was able to enhance MSC recruitment from a parabiotic source at the fracture site in a surgically conjoined mice pair model. The report also suggested the involvement of SDF-1/CXCR4 signaling pathway by an apparent increase immuno-detection of the two proteins [24].

In this study, we attempted to investigate that under LIPUS treatment, (a) the migration of MSCs to the fracture site; (b) the role of SDF-1/CXCR4 in regulating the recruitment of MSCs; (c) the MSCs engraftment and fracture healing. The aim of the first part of this study was to evaluate the direct influence of LIPUS on MSCs migration and intracellular SDF-1/CXCR4 signaling *in vitro*. The second part was to investigate the effects of LIPUS on MSCs recruitment and femoral fracture repair in a rat model, with or without blocking SDF-1/CXCR4 pathway.

## Materials and Methods

### 2.1. MSCs Isolation and Identification

#### 2.1.1. MSCs Isolation

All experiments were approved by the Animal Experimentation Ethics Committee (AEEC) of the authors’ institution (Ref: 10/007/GRF-5). MSCs were isolated from two 8-week female Sprague-Dawley (SD) rats, following protocol previously established in our laboratory [25]. Briefly, intact tibiae and femora were collected from euthanized healthy 8-week SD rats and carefully dissected free of muscles in the Petri dish containing sterile phosphate-buffered saline (PBS) and 1% penicillin-streptomycin (Invitrogen Corporation, Carlsbad, California, US). The bones were rinsed once in sterile 1×PBS before being transferred to the biosafety cabinet hood in the culture room. After rinsed once with sterile 1×PBS, the bone ends were cut with bone clipper. With the cut surface facing the bottom of the centrifuge tube, the tube was spun at 2000 rpm for 1 minute (most of the bone marrow (BM) was collected at the bottom of the tube). Mononuclear cells were then isolated by density gradient centrifugation (850 g, 30 minutes) using Lymphoprep (1.077 g/ml; AXIS-SHIELD PoC AS, Oslo, Norway), and re-suspended in complete culture medium containing alpha minimum essential medium (α-MEM) (Gibco, Grand Island, NY, US), 10% fetal bovine serum (FBS) (Gibco, Grand Island, NY, US), 100 U/ml penicillin, 100 µg/ml streptomycin and 2 mM L-glutamine (Invitrogen, Carlsbad, California, US). These mononuclear cells were plated at an optimal low cell density (10^5^ cells/cm^2^) to isolate stem cells and cultured at 37°C, 5% CO_2_/20% O_2_ to form colonies. When colonies reached 80–90% confluence, the MSCs were sub-cultured and re-plated for further expansion. Medium was changed every three days. Cells at passage 3 were used for all the experiments. The surface marker expression and multi-lineage differentiation potential of MSCs were characterized before being used for the further experiments.

#### 2.1.2. MSCs Characterization

The methods of the characterization of MSCs in this study were mainly based on the minimal criteria for human MSCs suggested by the Mesenchymal and Tissue Stem Cell Committee of the International Society for Cellular Therapy [26].

#### Flow Cytometry Assay

The surface marker expression of MSCs isolated from healthy rats, including CD90, CD44, CD45, CD31 and CD34, was analyzed by flow cytometry assay as described in the previous study [27]. Briefly, the MSCs at passage 3 were harvested by trypsinization, washed twice in PBS, then pelleted by centrifugation at 350 g for 5 minutes at room temperature and re-suspended in the staining buffer (Becton Dickson, Franklin Lakes, NJ, US) at 2×10^6^/ml for 15 minutes at 4°C. One-hundred microliters cell suspension was incubated with primary antibodies against rat CD90 and CD44 (Abcam, Cambridge, UK) conjugated with phycoerythrin (PE), CD31 (Abcam, Cambridge, UK) and CD34 (Santa Cruz Biotechnology, Santa Cruz, CA, US) conjugated with fluorescein isothiocyanate (FITC), CD45 (Abcam, Cambridge, UK) without conjugation for 15 minutes at 4°C. Unbound antibodies were washed away by adding ice-cold staining buffer. The cell pellet was re-suspended in the staining buffer containing goat anti-rabbit immunoglobulin G (IgG) conjugated with FITC (Santa Cruz Biotechnology, Santa Cruz, CA, US) for CD45 detection for at least 15 minutes at 4°C. The cells were washed with ice-cold PBS containing 2% bovine serum albumin (BSA) before analysis using the LSRFortessa flow cytometer (Becton Dickinson, San Jose, CA, US). PE-conjugated isotype-matched mouse IgG1 (R&D systems Inc, Minneapolis, MN, US) was used as isotype control for both CD90 and CD44; FITC-conjugated isotype-matched mouse IgG1 (R&D systems, Inc, Minneapolis, MN, US) was used as isotype control for both CD31 and CD34; and rabbit polyclonal IgG (Epitomics, Burlingame, CA, US) was used as isotype control for CD45.

#### Osteogenic Induction Assay and Alizarin Red S Staining

The methods have been described previously [25]. Briefly, MSCs at passage 3 were trypsinized and re-plated in a six-well plate at a concentration of 1×10^5^ cells per well, and cultured in complete culture medium for three days. These cells were then incubated in osteogenic induction medium (OIM) containing 100 nmol/L dexamethasone, 10 mmol/L beta-glycerophosphate, and 0.05 mmol/L L-ascorbic acid-2-phosphate. The OIM was changed every three days for 21 days. Cell and matrix layer was washed with PBS, fixed with 70% ethanol for 10 minutes, and stained with 0.5% Alizarin Red S (pH 4.1, Sigma, St. Louis, MO, US) for 30 minutes.

#### Adipogenic Induction Assay and Oil Red-O Staining

The method has been described previously [25]. MSCs were trypsinized and re-plated in a six-well plate at the same concentration as that for the osteogenic assay. The cells were cultured in the complete culture medium for three days, and were then incubated in adipogenic induction medium (AIM) containing 10% FBS, 1 µM dexamethasone, 10 µg/ml insulin, 50 µM indomethacin, and 0.5 mM isobuthyl-methylxanthine. The cells were cultured for an additional 21 days for assessment of the presence of oil droplets, which was confirmed by staining the cells with 0.3% fresh Oil Red-O solution (Sigma-Aldrich, St. Louis, MO, US) for 2 hours after fixation with 70% ethanol for 10 minutes.

### 2.2. LIPUS Treatment of MSCs

The isolated rat MSCs were divided into control (CG) or LIPUS treatment (UG) group (n = 5). The procedure of LIPUS treatment on cells was based on our previous protocol [19], [28]. Briefly, LIPUS was provided by a Sonic Accelerated Fracture Healing System (Smith & Nephew, Memphis, TN, US) for cell culture. The 6-well culture plate (Corning, Lowell, MA, US) was placed on the matched ultrasound transducers with a thin layer of coupling gel. The ultrasound energy was calibrated by the manufacturer and tested with the output checker before use. For UG, LIPUS treatment (unfocused plane waves, frequency 1.5 MHz, duty cycle 1∶4, spatial average-temporal average intensity 30 mW/cm^2^, pulse repetition frequency 1 kHz for pulse duration of 200 µs) was given from the bottom of the culture plate for 20 minutes/day in open air at 37°C for 3 days. CG received sham LIPUS treatment, with the culture plate placed on the transducers (with coupling gel) yet without ultrasound. On day 4, after changing the medium, the old conditioned medium from each group was collected for SDF-1 protein level analysis and cell migration assay; on day 7, the MSCs were harvested for real-time RT-PCR analysis.

### 2.3. Real-time RT-PCR Analysis

After 6 days of treatment, total RNA was isolated based on the established protocol [17]. The mRNA was then reverse-transcribed and amplified (Applied Biosystems, CA, USA). The synthesized cDNA was used as the template to quantify the relative content of mRNA by using LightCycler Real-Time PCR System (Roche Diagnostics, Penzberg, Germany). The glyceraldehyde 3-phosphate dehydrogenase (GAPDH) was used as the internal control. Primer sequences used in the experiments are summarized in Table 1 (all from Invitrogen, Carlsbad, CA, USA).

**Table 1:** Primer sequences of the target genes.

Gene	Primer nucleotide sequence	Product Size (bp)	Ta (°C)
SDF-1	Forward: 5′-TTGCCAGCACAAAGACACTCC-3′ Reverse: 5′-CTCCAAAGCAAACCGAATACAG-3′	225	58
CXCR4	Forward: 5′-TCCGTGGCTGACCTCCTCTT-3′ Reverse: 5′-CAGCTTCCTCGGCCTCTGGC-3′	210	56
GAPDH	Forward: 5′-AACTCCCATTCCTCCACCTT-3′ Reverse: 5′-GAGGGCCTCTCTCTTGCTCT-3′	200	57

GAPDH  =  glyceraldehide-3-phosphate dehydrogenase.

### 2.4. SDF-1 Protein Level Measurement

The culture medium was collected from UG and CG after 3 days of treatment. Protein level of SDF-1α was quantified using Quantikine SDF-1α enzyme immunoassay kit (R&D System, Minneapolis, Minnesota, USA). Colorimetric density of the developed plates was determined using BioTek µQuant Microplate Spectrophotometer (Bio-Tek Instruments Inc, Winooski, VT, US) at 450 nm wavelength. The enzyme-linked immunosorbent assay (ELISA) assay was performed in duplicate. A standard curve was constructed by plotting the mean absorbance for each standard against the concentration.

### 2.5. Cell Migration Assay

QCM 24-Well colorimetric cell migration assay (Millipore, MA, US) was used to study the migration of MSCs under LIPUS treatment, with or without the presence of AMD3100 (Sigma, St Louis, MO, US), in comparison with sham control [29]. AMD3100 is a specific antagonist of CXCR4 and not cross-reactive with other chemokine receptors [30]. MSCs at passage 3 were starved in serum-free medium for 1 day, washed twice with PBS and incubated in Harvesting Buffer at 37°C for 15 minutes. 20 ml Quenching Medium was added and cells were centrifuged at 1500 rpm for 5 minutes. The pellet was re-suspended in Quenching Medium, and brought to a final concentration at 5.0×10^5^ cells/ml. 300 µl cell suspension were added to each insert (8 µm pore size). [31] These inserts then were randomly divided into 3 groups (n = 5), including LIPUS treatment group (UG), LIPUS plus AMD3100 treatment group (UAG) and sham control group (CG). For UAG, an additional 1 µM AMD3100 was added to the insert. LIPUS treatment was applied with the same custom-built platform designed for 6-well plated as described above, then 500 µl of old serum-free conditioned medium collected after 3 days of LIPUS treatment were added to the lower chambers in UG and UAG; 500 µl of old serum-free conditioned medium collected after 3 days of sham treatment was added to the lower chamber in CG. MSCs were incubated at 37°C in 5% CO_2_/20% O_2_ for 18 hours. The remaining cell suspension was removed; the migration insert was placed into a clean well containing 400 µl of Cell Stain and incubated for 20 minutes at room temperature. The insert was rinsed in water several times, and non-migratory cells were carefully removed from the interior of the insert. The migrated cells on the exterior of the insert were counted manually under the microscope (Leica DM IRB, Heerbrugg, Switzerland), and the images were taken at ×100 magnification.

### 2.6. Animal Model, Groupings and LIPUS Treatment

Thirty 8-week-old female SD rats were obtained from the Laboratory Animal Services Center of the Chinese University of Hong Kong. Closed femoral fractures were created at femur shaft based on our established protocol [32], [33]. Postoperative radiographies were used to confirm the quality of fracture.

On day 3 post-fracture, the rats were anaesthetized by intraperitoneal injection of a mixture of ketamine (50 mg/kg) and xylazine (10 mg/kg). 1.0×10^6^ GFP-labelled MSCs (RASMX-01101, Cyagen, Guangzhou, China) in 500 µl PBS were transplanted by intracardiac injection as previous described [34], [35]. Briefly, the GFP-labeled MSCs were sub-cultured to the fifth passage, and then were trypsinized and diluted in 0.9% normal saline. Under anaesthesia, the rat was placed supine, kept firmly on the desk holding the chest between the thumb and forefinger. After removing the air in the liquid, a 23G needle was inserted through the thoracic wall at a point left to the sternum on a line connecting the left axillary pivot with the caudal tip of the sternum. In the meantime, the syringe containing MSCs suspension was aspirated; the injection of the MSCs suspension was performed by gently pressing the piston of the syringe. The position of needle was confirmed by an ultrasound system (Vevo 770, VisualSonics, ON, Canada) under B-mode (brightness mode).

After MSCs injection, the rats were randomly assigned to the following groups: 1) LIPUS group (UG, n = 10) in which LIPUS treatment (Exogen 3000+, Smith & Nephew Inc, Memphis, TN, USA) was applied 20 minutes/day, 5 days/week, after the cell injection. During treatment, the rats were laid on the ventral side under general anaesthesia and a 2.5 cm diameter ultrasound transducer was placed on the lateral side of the fracture site. The ultrasound signal consisting of a 200 µs burst of 1.5 MHz sine wave repeating at 1.0 kHz with 30.0±5.0 mW/cm^2^ spatial average and temporal average incident intensity was given. 2) LIPUS+AMD3100 group (UAG, n = 10) in which daily LIPUS treatment was applied (same as UG), AMD3100 was resolved in saline to a final concentration of 1 mg/ml for injection and administered (1 mg/kg/day, intraperitoneal) [36] 30 minutes before LIPUS treatment. 3) Sham control group (CG, n = 10) in which the daily sham treatment (LIPUS machine turned off) was applied. Both UG and CG groups received vehicle injections of 0.9% normal saline (1 ml/kg/day, intraperitoneal). Animals were allowed full weight bearing, free cage activity, and food and water ad libitum. At week 4 post-fracture, animals were euthanized by overdosed sodium pentobarbital; the femora and blood were collected for the end-point assessments.

### 2.7. Radiological Assessment

Two-dimensional digital radiographs (MX-20, Faxitron, Lincolnshire, IL, USA) were taken weekly post-fracture to confirm the quality and degree of fracture healing. The quantified temporal changes of callus morphology were evaluated by using the Metamorph Image Analysis System (Universal Imaging Corporation, Downingtown, PA, USA) according to our previous established protocol [37], where callus width (CW) was defined as the maximal outer diameter of the mineralized callus (d2) minus the outer diameter of the femur (d1); and callus area (CA) was calculated as the sum of the areas of the external mineralized callus.

### 2.8. Ex Vivo GFP Signal Intensity Analysis

Animals were euthanized at week 4 post-fracture. The *ex vivo* fluorescent images were taken by the Xenogen Imaging System (IVIS 200; Caliper Life Sciences, Hopkinton, MA, USA), immediately after the femur was harvested with removal of soft tissues and K-wire. The GFP signals at callus area were acquired and measured by using the live image 2.5 software of Xenogen Imaging System with the settings of exposure time at 5 seconds, binning at 8 and f/stop at f/16 [12], [35]. A standard circular region of interest (ROI) with a diameter of 1 cm at the callus area was selected for the measurements. The fluorescent image data was displayed in units of photons. The tissue autofluorescence was subtracted by using GFP background filter with the excitation passband at 440 nm, and emission passband at 550 nm.

### 2.9. µCT Analysis

*Ex vivo* micro-computed tomography scans (µCT40, Scanco Medical, Brüttisellen, Switzerland) were performed at 4 weeks post-fracture based on our established protocol [32], [38]. The femora were positioned vertically with normal saline-soaked gauze in the sample holder during scanning. The newly formed bone (low-density bone, threshold = 165–350) and highly mineralized bone (high-density bone, threshold = 350–1000) were reconstructed separately [16], [32]. The ratios of low-density bone volume over total tissue volume (BVl/TV), high-density bone volume over total tissue volume (BVh/TV), total bone volume fraction (BVt/TV) and bone mineral density (BMD) were calculated.

### 2.10. Mechanical Testing

A complete healing of closed femoral fracture in young rat was reported taking place around week 4 post-surgery [39]. After µCT scanning, the fractured femora were subjected to mechanical testing as previously described [32], [37]. The ultimate load (UL), stiffness, and the energy to failure were calculated from load displacement curves using built-in software (QMAT Professional Material testing software).

### 2.11. Histomorphometric and Immunohistochemical Analysis

The harvested samples were performed hematoxylin–eosin (H&E) and safranin-O/fast green staining for histomorphometric analysis. The images were taken at ×50 magnification under microscope (Leica DMRB DAS; Leica, Heerbrugg, Switzerland). Quantitative assessment of the safranin-O-positive cartilage at the region of 1.5 mm proximal and distal to the fracture line was performed by using ImageJ (NIH, MD, USA). Cartilage area (Cg.Ar), callus area (Cl.Ar) and their ratio (Cg.Ar/Cl.Ar) were measured.

Immunohistochemical staining was carried out on deparaffinized sections using a rabbit ABC staining system (Santa Cruz Biotechnology, Santa Cruz, CA, USA). The sections were incubated overnight at 4°C in 1∶500 rabbit anti-GFP polyclonal antibody (Abcam, Cambridge, MA, USA) or 1∶200 rabbit anti-SDF-1 polyclonal antibody (Abcam, Cambridge, MA, USA), followed by incubation with biotinylated secondary antibody and color development according to the manufacturer’s instructions. Images were captured using bright field microscopy at ×100 and ×200 magnification (Leica DMRB DAS; Leica, Heerbrugg, Switzerland).

### 2.12. Blood Collection and Serum SDF-1 Analysis

**Figure 1:**
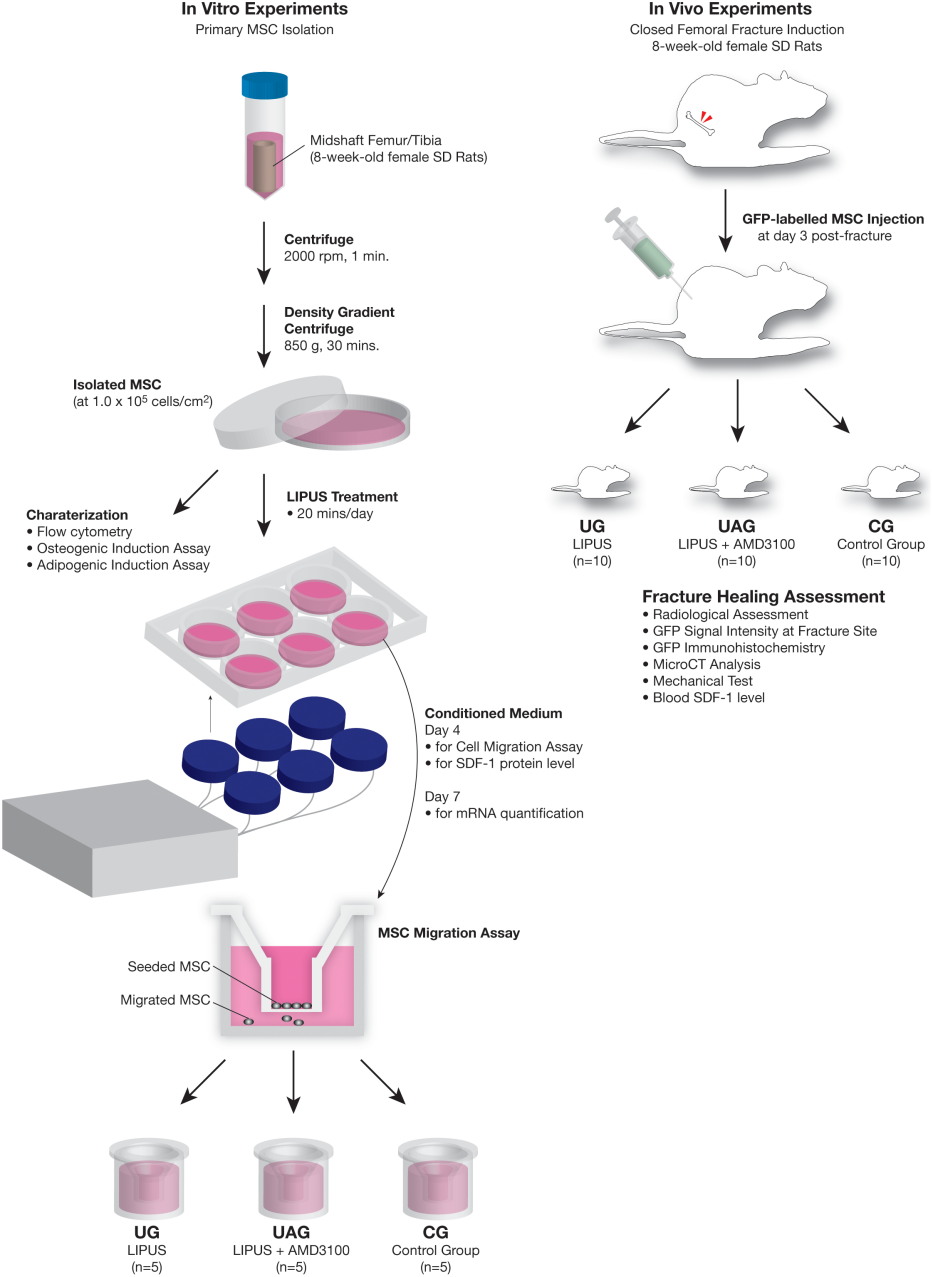
Flowchart of the study design. MSCs were isolated from two 8-week-old female SD rats and characterized by flow cytometry assay, osteogenic induction assay and adipogenic induction assay. In the in vitro experiments (n = 5), the SDF-1 protein and mRNA expression levels in condition medium were compared between control (CG) and LIPUS treatment (UG) groups, the MSCs migration ability was compared among CG, UG and LIPUS plus AMD3100 treatment (UAG) groups. In the in vivo experiments, closed femoral fractured rats were randomly divided into CG, UG and UAG groups (n = 10). GFP-labeled MSCs were intracardiac injected to all the rats on day 3 post-fracture and recruitment effects by LIPUS were compared among groups.

Five milliliter of blood was collected by cardiac puncture shortly before the animals were euthanized. The blood was centrifuged at 1,800 g for 10 minutes, and the separated serum samples were then stored at −80°C until analysis. The level of SDF-1 in the serum was measured by using the same SDF-1α ELISA kit and microplate reader settings used for culture medium testing as described above. All the serum samples were run in duplicate. A flowchart of the study design was shown in Fig. 1.

### 2.13. Statistical Analysis

All quantitative data were expressed as mean ± standard deviation and analyzed with SPSS version 20.0 software (IBM, NY, USA). Independent student’s *t* test and one-way analysis of variance (ANOVA) with Tukey’s post-hoc test were used for two-group or multiple-group comparisons respectively, as time since fracture induction was not considered as independent variable due to known temporal changes for our measured parameters. Statistical significance was set at p<0.05.

## Results

### 3.1. MSCs Identification

**Figure 2:**
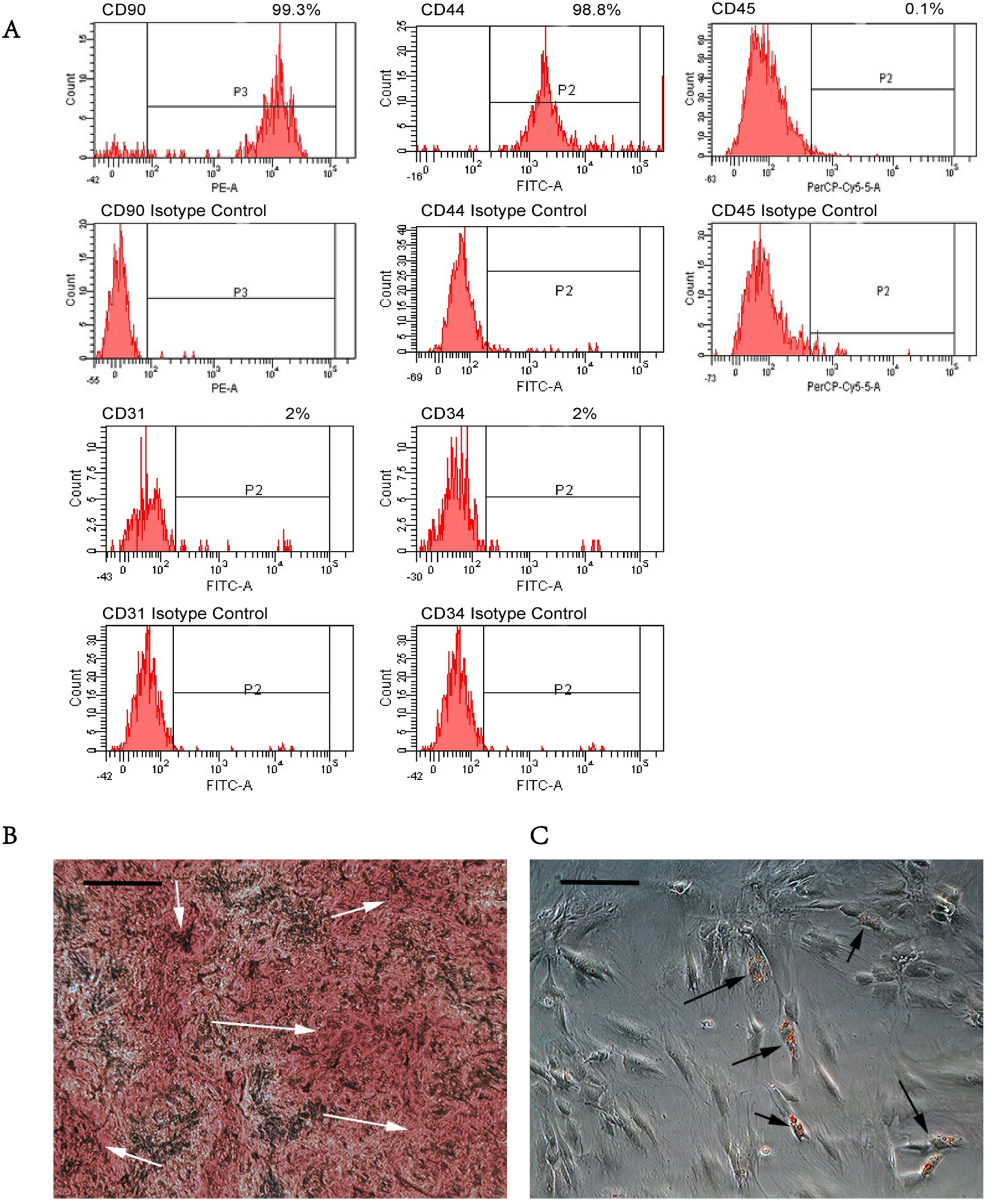
MSCs characterization. (**A**) The expressions of selected surface markers on the isolated cells from BM of SD rats. This figure shows the expressions of mesenchymal stem cell markers (CD90 and CD44), endothelial cell marker (CD31) and hematopoietic cell markers (CD34 and CD45) on the isolated cell colonies. (**B**) Representative microphotograph of Alizarin Red S-stained cells isolated from BM of SD rats. White arrows indicate the obvious calcium deposition areas in the matrix. Scale bar = 100 µm. (**C**) Representative microphotograph of Oil Red O-stained cells isolated from BM of SD rats. The black arrows indicate the adipose-differentiated cells. The small red bubbles in cells are lipids. Scale bar = 100 µm.

The results of flow cytometry demonstrated that over 99.3% and 98.8% of the mononucleated cell colonies isolated from BM of SD rats were positive for the fibroblastic marker CD90 and MSC marker CD44 respectively. They were negative for the endothelial stem cell marker CD31 and negative for the hematopoietic lineage markers, including CD34 and CD45 (Fig. 2A). Osteogenic induction assay showed abundant calcium deposits in the cell culture (Fig. 2B). Adipogenic induction assay indicated that a number of isolated cells were positively stained by Oil Red O (Fig. 2C).

### 3.2. Real-time RT-PCR and SDF-1 Protein Level Measurement

**Figure 3:**
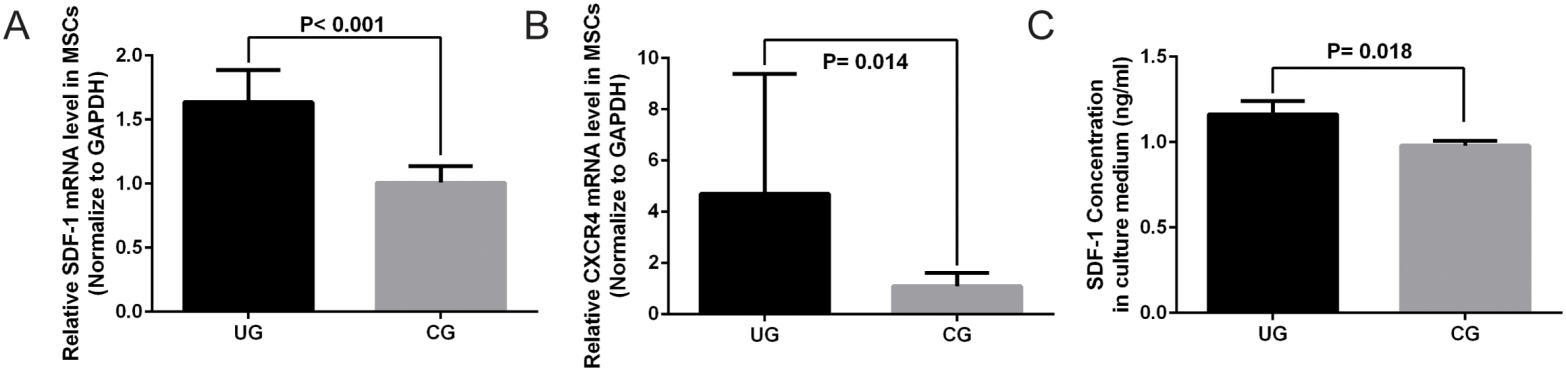
Target genes quantification and SDF-1 protein measurement. (**A**) LIPUS enhanced SDF-1 gene expression in MSCs significantly (p<0.0001), and (**B**) LIPUS enhanced CXCR4 gene expression in MSCs significantly (p = 0.014). (**C**) ELISA assay showed increased SDF-1 protein level in the culture medium of MSCs treated with LIPUS (p = 0.018).

Real-time RT-PCR and ELISA results demonstrated that gene expression of SDF-1 and CXCR4 were significantly upregulated in UG, as compared with CG (p<0.0001 and p = 0.014, respectively) (Fig. 3A, B). The SDF-1 and CXCR4 mRNA levels were increased 1.6 times and 4.3 times in UG than in CG respectively. SDF-1 protein level in the culture medium was also significantly increased in UG than CG (p = 0.018) (Fig. 3C).

### 3.3. Cell Migration Assay

**Figure 4:**
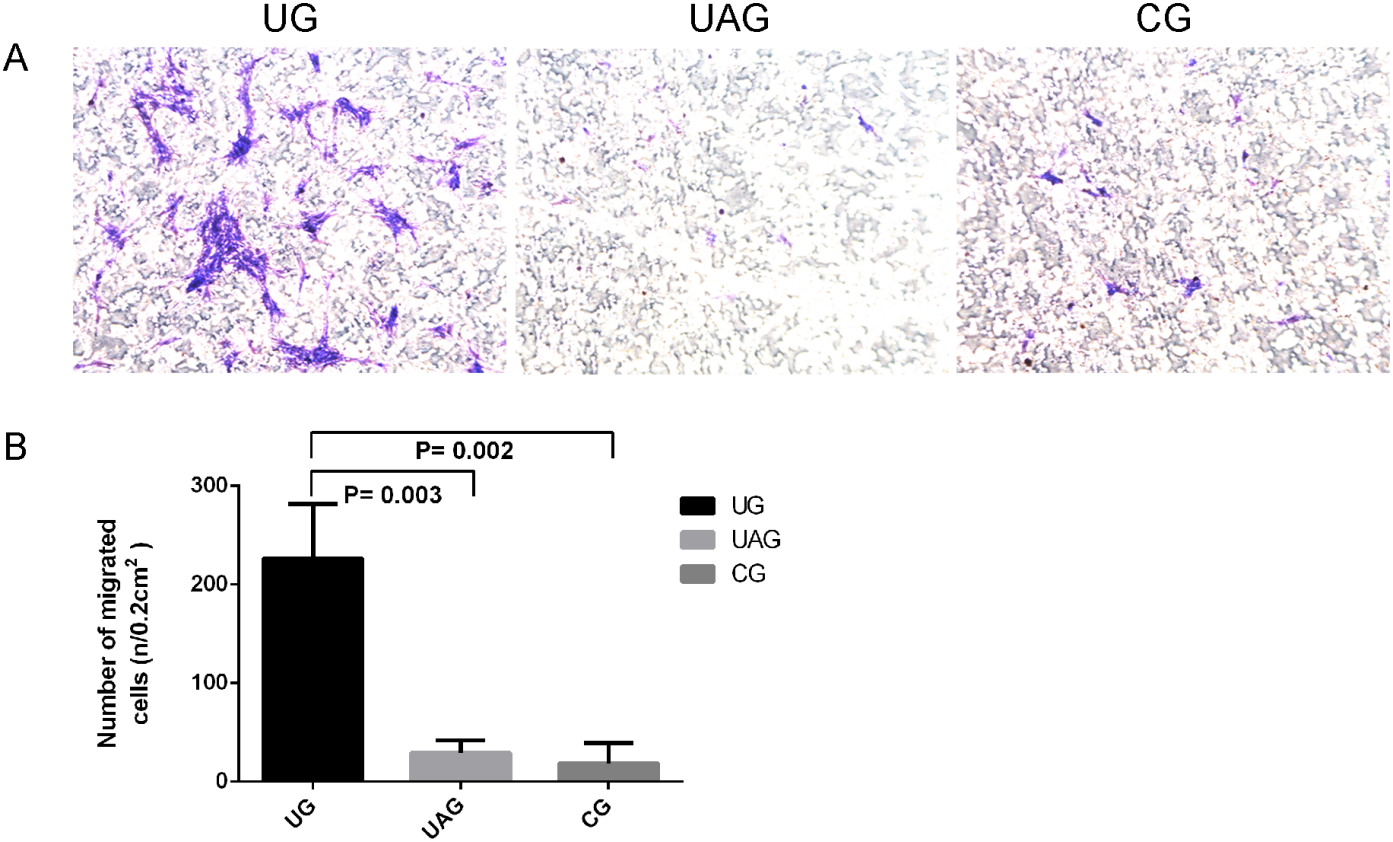
MSCs migration assay. (**A**) The migrated MSCs on the exterior of the insert in UG (left) were remarkably increased compared with UAG (middle) and CG (right) at ×100 magnification. (**B**) The number of migrated cells in UG was increased 12.1 times than in CG (p = 0.002); the number of migrated cells in UAG was decreased by 87.2% as compared to UG (p = 0.003).

Under light microscopy, abundant MSCs were found on the exterior of the inserts in UG (Fig. 4A, left); whereas only a small number of MSCs were observed on the exterior of the inserts in both UAG (Fig. 4A, middle) and CG (Fig. 4A, right).

Quantitatively, the number of migrated MSCs in UG was significantly increased than in UAG and CG (p = 0.003, p = 0.002, respectively) (Fig. 4B). No significant difference was observed in the number of migrated cells between UAG and CG.

### 3.4 Radiological Assessment

**Figure 5:**
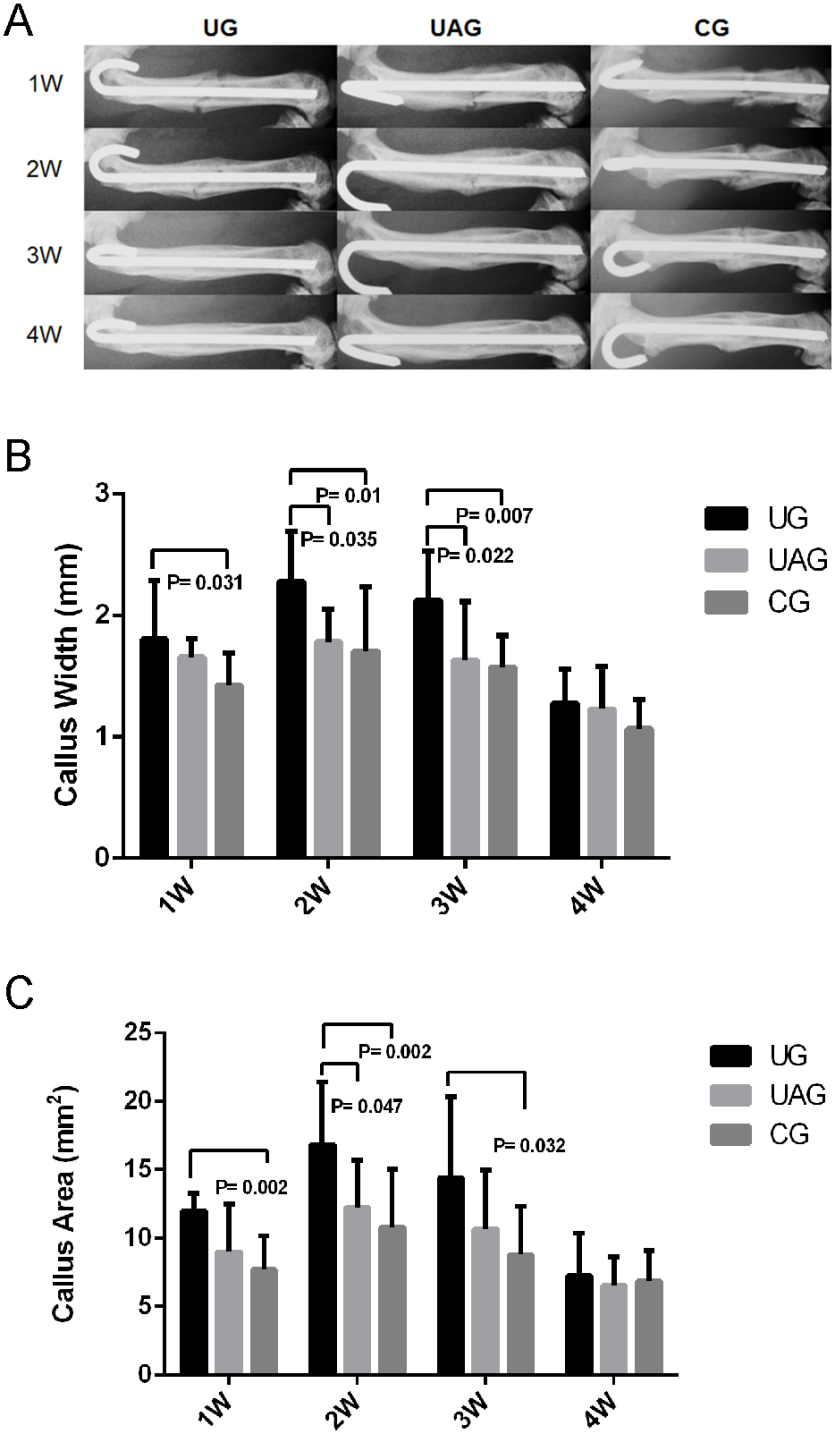
Radiographic analysis of fracture healing in young rats. (**A**) Series of representative radiographies showed better callus bridging in UG and UAG, compared with in CG at different time points. (**B**) The measurement of callus width (CW) showed: CW in UG was larger by 26.8% at week 1 (p = 0.031), by 33.6% at week 2 (p = 0.01) and by 35.0% at week 3 (p = 0.007) post-fracture than in CG, and by 27.8% at week 2 (p = 0.035) and by 30.0% at week 3 (p = 0.022) post-fracture than in UAG. (**C**) The quantitative measurement of callus area (CA) showed: CA was significantly larger in UG by 55.1% at week 1 (p = 0.002), by 55.5% at week 2 (p = 0.002), by 64.0% at week 3 (p = 0.032) than in CG, and was significantly larger by 37.7% at week 2 than in UAG (p = 0.047).

Radiographic analysis demonstrated that both UG and UAG showed earlier fracture healing than CG, as indicated by earlier callus bridging occurred at week 2, whereas callus bridging started from week 3 in CG (Fig. 5A). Quantitative measurement of callus morphometry showed that CW was significantly larger in UG than in CG at week 1, 2 and 3 (p = 0.031 for week 1, p = 0.01 for week 2, p = 0.007 for week 3, respectively); CW was significantly larger in UG than in UAG at week 2 (p = 0.035) and week 3 (p = 0.022) (Fig. 5B). CA was significantly larger in UG than in CG at week 1, 2 and 3 (p = 0.002 for week 1, 2; p = 0.032 for week 3); CA was significantly larger in UG than in UAG at week 2 (p = 0.047) (Fig. 5C).

### 3.5. Ex Vivo GFP Signal Intensity Analysis

**Figure 6:**
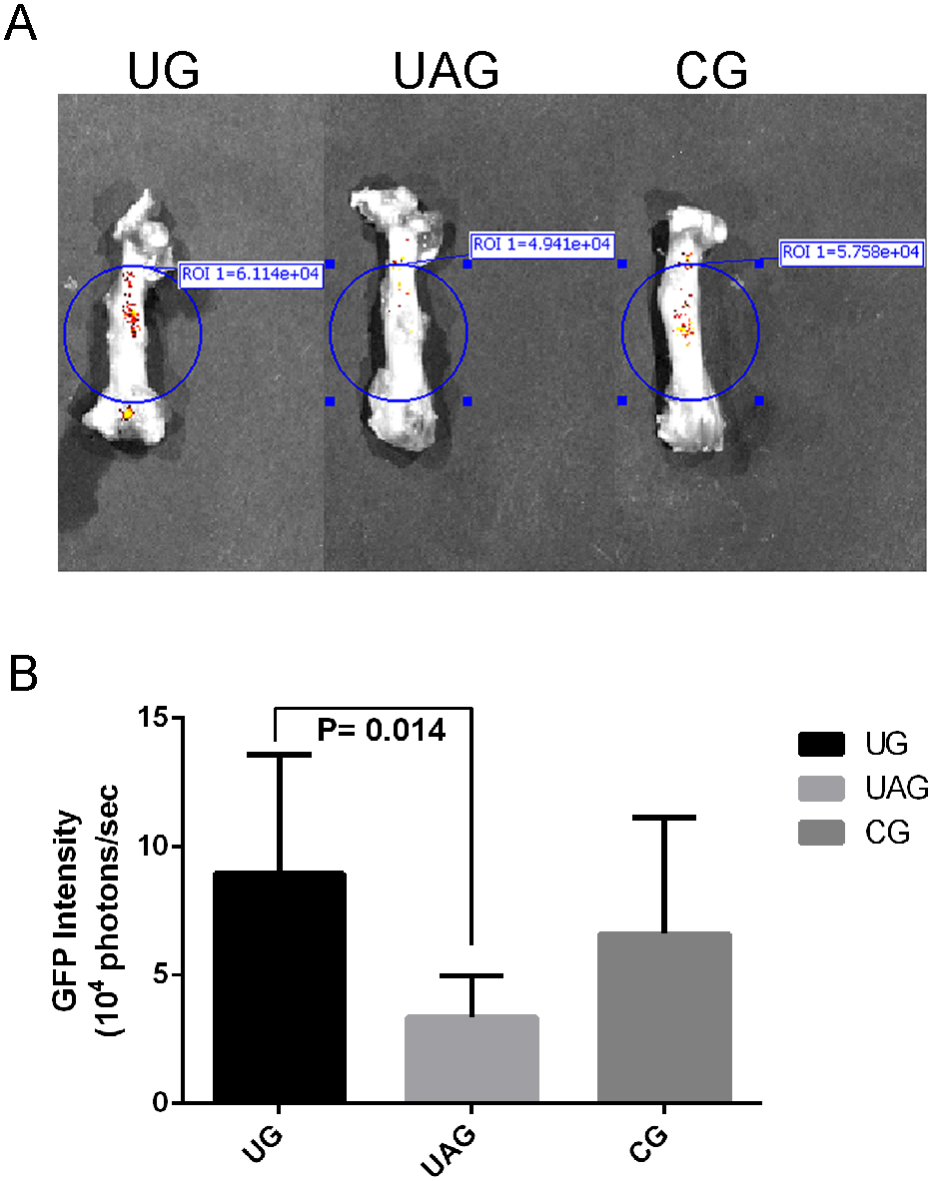
*Ex vivo* GFP intensity measurement. (**A**) On the representative image of each group, blue circles indicate the region of interest (ROI) for fluorescent imaging analysis. GFP signals in UG (left) was much higher than in UAG (middle) and CG (right). (**B**) Semi-quantitative GFP intensity of fracture callus in UG was increased 2.66 times than in UAG (p = 0.014), although no significant differences were found among other groups.

GFP intensity in UG was significantly higher than in UAG (p = 0.014). Higher GFP intensity was found in UG as compared with CG, and in CG as compared with UAG, but no significance was found between these groups (Fig. 6).

### 3.6. µCT Analysis

**Figure 7:**
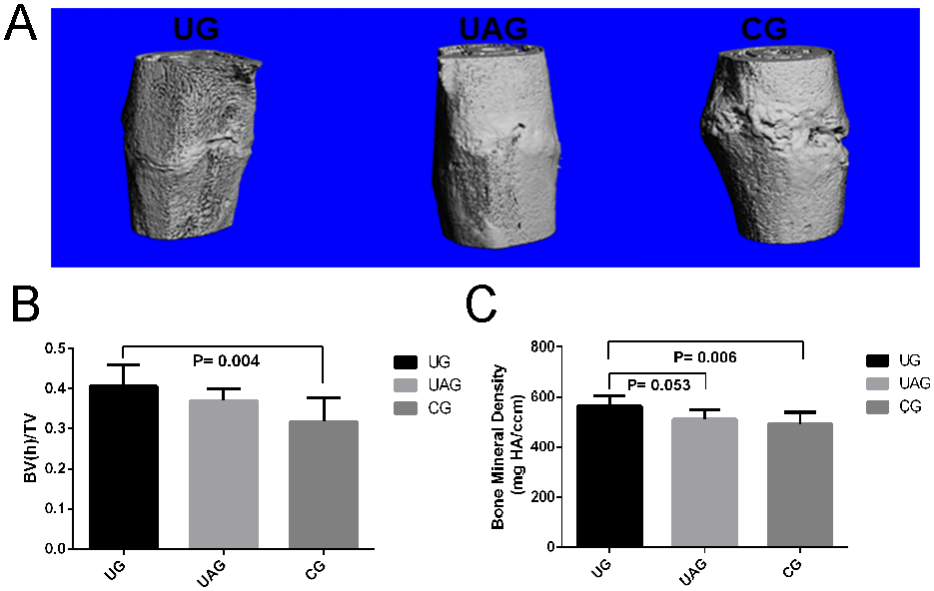
µCT measurement. (**A**) Representative 3D reconstructed micro-CT images of the three groups at week 4 post-fracture showed improved fracture healing in UG and UAG. (**B**) BVh/TV in UG was increased by 27.9% than in CG (p = 0.004). (**C**) BMD in UG was higher by 10% than in UAG (p = 0.053) and by 14.5% than in CG (p = 0.006).

3D reconstructed µCT images of the three groups at week 4 post-fracture showed that the fracture healing in UG and UAG were much faster than in CG, as indicated by early closure of fracture gap (Fig. 7A). The BVh/TV in UG was significantly higher than in CG (p = 0.004) (Fig. 7B); BMD in UG was higher than in UAG (p = 0.053) and CG (p = 0.006) (Fig. 7C). For BVl/TV and BVt/TV, no significant differences were found among groups.

### 3.7. Mechanical Testing

Femora in UG were significantly stronger than in other two groups (Table 2). The ultimate load of the fractured femur in UG was significantly greater than those in UAG and CG (p<0.05, p<0.05 respectively). The stiffness of the fractured femur in UG was also marginally higher than that of CG (p = 0.065).

**Table 2:** Mechanical properties of the femurs of the three groups at week 4 post-fracture.

Parameters	UG	UAG	CG
Ultimate Load (N)	126.2±7.0^a^^,^ b	96.9±10.4^c^	62.9±26.9
Stiffness (N/mm)	53.5±7.2^b^	44.9±12.4	38.7±10.9
Energy to Failure (j)	0.05±0.02	0.03±0.01	0.03±0.02

UG, LIPUS treatment group; UAG, LIPUS+AMD3100 treatment group; CG, control group received sham treatment.

a p<0.05 between UG and UAG;

b p<0.05 between UG and CG;

c p<0.05 between UAG and CG.

### 3.8. Histomorphometric and Immunohistochemical Analysis

**Figure 8:**
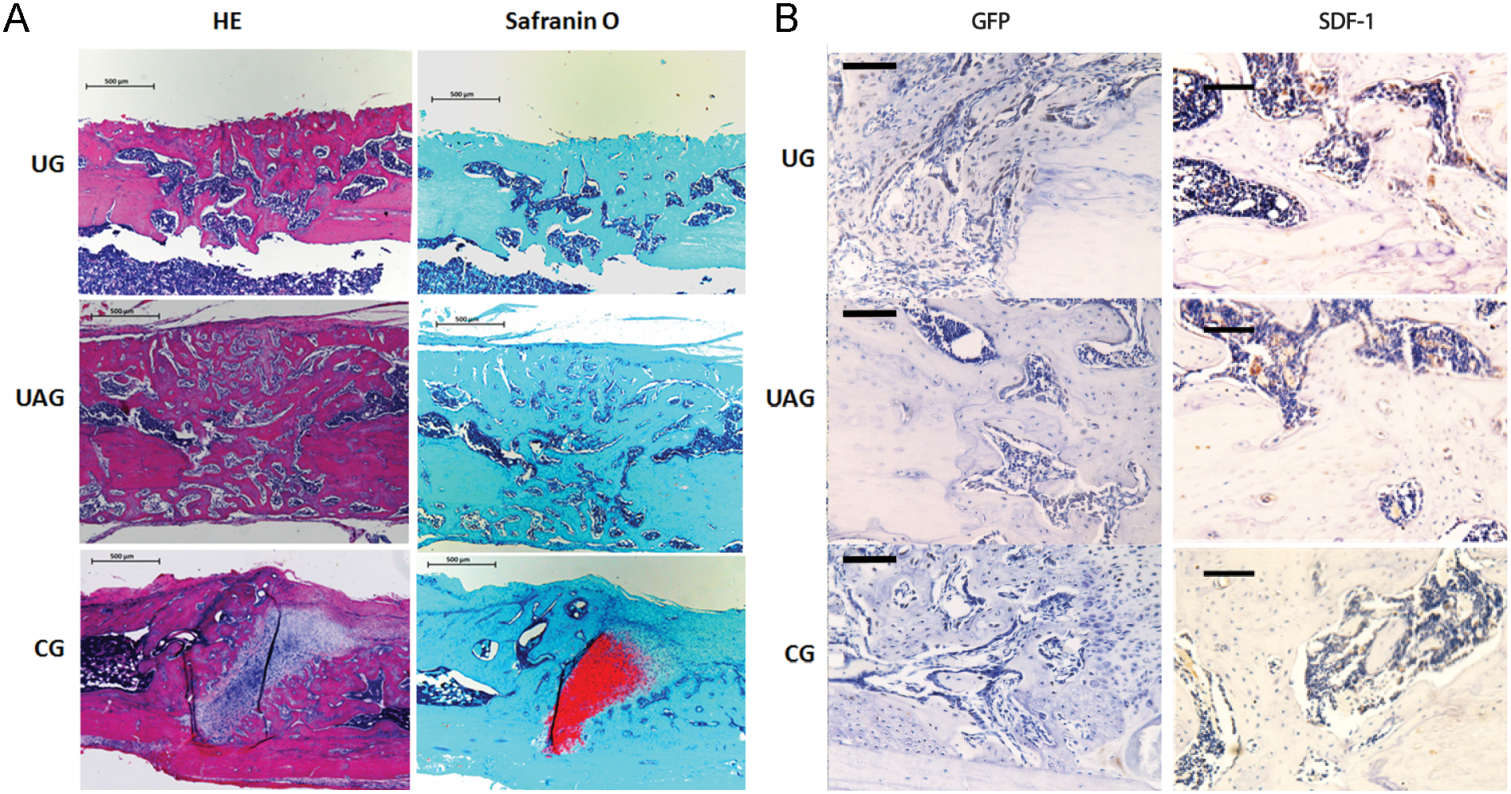
Histological and immunohistochemical results. (**A**) Representative H&E and safranin-O/fast green staining showed that in UG and UAG, there were large amounts of woven bone formation in the fracture areas, with almost no chondroid tissues (stained red by safranin-O), whereas lots of chondroid and fibrous tissues still existed at the fracture site in CG at week 4. Scale bars, 50 µm. (**B**, left) Representative immunohistochemistry for GFP showed that a large number of the GFP positive cells engrafted in the fracture area in UG, whereas fewer GFP positive cells were detected in CG, and even fewer GFP positive cells were found in UAG. Scale bars at ×100, 200 µm. (**B**, right) Representative images of SDF-1 staining of callus at week 4 post-fracture for young rats. Brown color indicates positive staining. SDF-1 was located mainly in the blood vessels or sinusoid-like regions. Scale bars at ×200, 100 µm.

Histological evaluation using hematoxylin–eosin (H&E) and safranin-O/fast green staining demonstrated enhanced fracture healing in UG and UAG at week 4, as reflected by newly formed woven bone with better bridging of fracture gap, whereas newly formed woven bone was observed to some extent in CG at week 4 but there were still many chondroid tissues in the fracture area (Fig. 8A). Quantitative analysis showed that Cg.Ar/Cl.Ar (%) in UG was significantly lower than in CG (Table 3).

**Table 3:** Histomorphometric analysis of femoral microarchitecture at week 4 post-fracture.

Parameters	UG	UAG	CG
Cl.Ar (mm^2^)	5.0±0.6	4.0±1.9	5.3±2.7
Cg.Ar (mm^2^)	0.0±0.0	0.1±0.0	0.9±0.9
Cg.Ar/Cl.Ar (%)	0.0±0.0^a^	0.0±0.0	0.1±0.1

UG, LIPUS treatment group; UAG, LIPUS treatment plus AMD3100 treatment group; CG, control group received sham treatment, Cl.Ar, total callus area; Cg.Ar, cartilage area; Cg.Ar/Cl.Ar, the percentage of cartilage area.

a p = 0.038 between UG and CG.

Immunohistochemical analysis of GFP and SDF-1 demonstrated that at week 4 post-fracture, many GFP positive cells could be found in the callus area in all three groups, where the number of GFP positive cells in UG was remarkably increased than those in UAG and CG. In UAG, only a scarce number of GFP-positive cells could be found in the fracture area, as compared with UG and CG (Fig. 8B, left). SDF-1 protein in the callus area of UG and UAG was higher than in CG respectively (Fig. 8B, right).

### 3.9. Serum SDF-1 Protein Level Measurement

**Figure 9:**
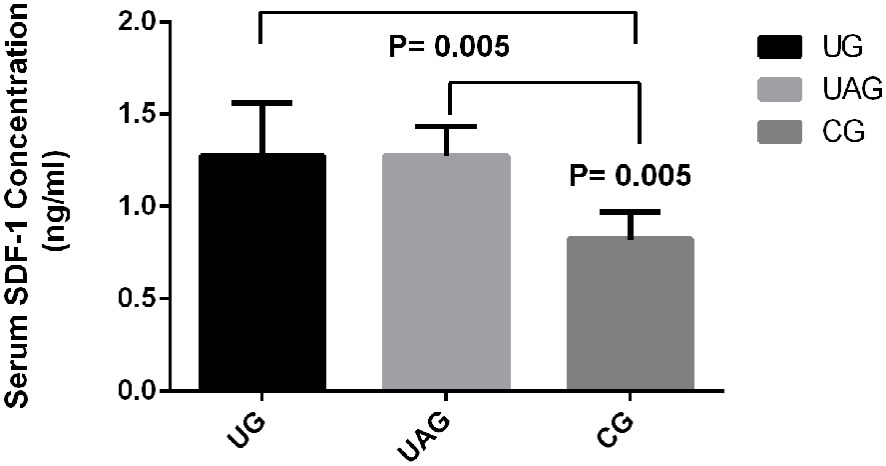
Serum SDF-1 protein concentration. The serum SDF-1 protein level measured by ELISA assay in UG was increased by 1.55 times than in CG (p = 0.005); the serum SDF-1 level in UAG was increased by 1.55 times than in CG (p = 0.005).

At week 4, the serum SDF-1 protein levels in UG and UAG were significantly increased, as compared with CG (p = 0.005 for both), while the level of SDF-1 in serum was comparable between UG and UAG (Fig. 9).

## Discussion

Fracture healing is a complex and well-orchestrated biological process composed of three phases: inflammation, repair and remodeling. In the inflammation and repair phases, SDF-1/CXCR4 signaling participates in bone repair mainly by acting as a regulator of MSCs trafficking to fracture site [40]. In this study, we confirmed that LIPUS applied on the fractured bone promoted MSCs recruitment and that SDF-1/CXCR4 played a very important role in this process. Blocking of SDF-1 signaling with AMD3100 inhibited the migration of MSCs, and also reduced the promoting effect of LIPUS on fracture healing. The present study demonstrates that the enhanced MSCs migration mediated by SDF-1/CXCR4 pathway may be one of the crucial mechanisms through which LIPUS promotes fracture healing.

A major finding of the current study is that physical stimulation in form of LIPUS, can promote MSCs migration *in vitro* and during bone fracture healing *in vivo*. Mechanical stimuli are very important for the development and maintenance of bone [41]. Recently, increasing studies have shown that mechanical stimulation is crucial for regulating MSCs activities during bone repair. Luu *et al*. reported that low magnitude mechanical signals could significantly increase the proliferation and osteogenic differentiation of MSCs in the bone marrow of male C57BL/6J mice [42]; Lai *et al*. further demonstrated that LIPUS could increase osteogenic differentiation of human MSCs [43]. However, the role of mechanical signals in regulating MSCs migration is not reported. Our results revealed that mechanical signals might work in several ways to regulate MSCs behavior and functions. From *in vitro* study, the spontaneous migration capacity of the isolated rat MSCs in CG was observed. Similar phenomenon was observed by Adriana *et al.* in an *in vitro* migration study, which demonstrated a low spontaneous migration capacity of BM-derived MSCs in the presence of medium alone (without growth factors or chemokines), after overnight incubation of the transwells at 37°C, 5%CO_2_ [44]. It was most likely that the conditioned medium in the lower chamber of CG contained many bio-active factors that served as chemoattractant and induced the spontaneous migration of MSCs [45]–[48]. Our data found that LIPUS stimulation enhanced the migration of cultured MSCs, as indicated by the increased number of migrated cells at the exterior of inserts from UG, compared with those in CG. The effect of LIPUS on cell migration has been reported by several studies. Takao *et al*. studied the migration of osteoblast-like cell (MC3T3-E1 cells) under LIPUS treatment by using a wound healing assay. They found that after 20 minutes LIPUS treatment, the migration of MC3T3-E1 osteoblastic cells was significantly increased than the control group, as indicated by the distance between the wound line and the migration front at 6 h, 12 h and 20 h after wounding [49]. The upregulated SDF-1 and CXCR4 expression by LIPUS may be responsible for the enhanced motility of MSCs observed in cell migration assay in two possible ways. First, the enhanced CXCR4 expression of MSCs could lead to the increased MSCs migration. Shyam *et al*. isolated and cultured MSCs from healthy volunteers and transduced them with a retroviral vector containing either CXCR4 and GFP or GFP alone. They used a transwell migration system to study MSC migration to SDF-1, and found that MSCs transduced with CXCR4 showed significantly more migration toward SDF-1, with 3-fold greater at 3 h and more than 5-fold greater at 6 h [50]. Second, the upregulated SDF-1 expression, especially the increased SDF-1 protein level in the conditioned medium, promoted MSCs migration. Previously Son *et al*. used a chemoinvasion assay to evaluate the ability of MSCs to cross the reconstituted basement membrane Matrigel. After 24 hours of incubation at 37°C, 5% CO_2_, the number of migrated MSCs toward the lower compartment containing SDF-1 was significantly higher than that in control [51]. Our results indicated that the enhanced cell migration may be due to the combined effects as described above. BM-derived MSCs are able to express CXCR4 and secrete SDF-1 simultaneously. The present study further demonstrated that after treating MSCs with AMD3100, the antagonist of SDF-1/CXCR4 pathway, the migration of MSCs under LIPUS treatment was strikingly reduced. This indicates the LIPUS-induced MSC migration is CXCR4-dependent. Although we still found a very small number of migrated cells in the combined treatment group (UAG), when compared with that in CG, there was no statistical significance. It suggested that AMD3100 might almost completely block the effect of LIPUS on MSC migration, since SDF-1 may not be the only chemokine in the conditioned medium of MSCs. Other bioactive factors secreted by MSCs may also influence MSC migration to some extent [45]–[48]. *In vivo*, the transplanted GFP-labeled MSCs were found to migrate to the callus, as indicated by the GFP intensity measured by fluorescent imaging and immunohistochemistry. Our findings confirmed that in young rat model, after 4 weeks of LIPUS treatment, both the serum and local SDF-1 protein levels in the callus of UG were increased than in CG, together with the higher GFP intensity from *ex vivo* fluorescent imaging, and increased GFP-positive cells in the callus of UG as detected by immunohistochemistry. Our findings were also substantiated by a similar report by Kumagai *et al.* demonstrating a positive effect in the recruitment of GFP-positive cells from a parabiotic mouse to the fracture site of another surgically conjoined mouse [24]. Therefore, there are sufficient evidence to conclude that there exist a strong relationship between LIPUS stimulation and MSCs migration.

This study demonstrated LIPUS treatment could activate SDF-1/CXCR4 pathway, which were substantiated by a few previous studies. Carlet *et al*. observed an intense expression of SDF-1 in the compression side of periodontal ligament during orthodontic tooth movement [52]; Li *et al.* also reported that cyclic stretch could upregulate SDF-1/CXCR4 axis in human saphenous vein smooth muscle cells [53]. Kumagai et al. also detected increased protein expression of both SDF-1 and CXC-R4 in the fracture site in the LIPUS treatment group as compared to the control group [24]. However, the mechanisms responsible for mechanical stimuli induced SDF-1/CXCR4 signaling in these cells are largely unknown. Integrins are the main receptors that connect the cytoskeleton to the extracellular matrix (ECM) [54]. They transmit mechanical stresses across the plasma membrane that enables the tractional forces developed in the cytoskeleton to be conveyed to the ECM [55]. Integrins also regulates signaling pathways [56], [57]. Many recent studies have demonstrated the important interactions between SDF-1/CXCR4 pathway and integrins in regulating cellular activities in different cell types, including MSCs [58]–[61]. Thus, the effect we observed might be the downstream of LIPUS’s interactions with the transmembrane integrins on the MSCs.

The direct regulatory effect of LIPUS on MSCs found *in vitro* may not fully reflect the complexity of the *in vivo* situation. During fracture repair, SDF-1 was found not only in MSCs, but also in other cell types, including endothelial cells [62], [63], periosteal cells [28], [64], chondrocytes [65], and osteoblasts [66], [67] etc. Many previous studies have shown these cells might also secrete SDF-1 and contribute to the recruitment of MSCs. Given the fact that mechanical signals provided by LIPUS could be sensed and transduced by many cell types, which has been extensively studied in the past [68]–[73], it is most likely that LIPUS may act on these cells through different ways. LIPUS might promote SDF-1 secretion from different cells through physical interactions [74], [75] and integrins signal transductions [76]–[79] in the site of injury; simultaneously, LIPUS enhances CXCR4 expression on the surface of MSCs from circulation or the adjacent BM, thus promoting these cells to migrate toward the SDF-1 gradient and engraft in the fracture site.

In the rat model, LIPUS promoted early callus formation, as indicated by weekly radiographic analysis. In all the groups, the temporal changes of CW and CA followed the similar pattern, i.e. from week 1 to week 2, both CW and CA increased gradually; from week 2 to week 4, both CW and CA decreased rapidly. The largest callus size was generally found at week 2, which indicated the most active callus formation; whereas the smallest callus size was found at week 4, which represented the callus remodeling. Significantly increased callus size in UG, in comparison with CG, was observed from week 1 to week 3 post-fracture, which reflected the promoting effect of LIPUS on callus formation in the early phase of fracture healing. Another finding of the radiographic analysis was that the callus bridging in UG was accelerated, which started at week 2, in contrast to CG at week 3. The present findings were consistent with many previous researches. In 1983, Dyson *et al*. applied therapeutic ultrasound on the complete bilateral transverse fibular fractures in adult female Wistar rats, and found that ultrasound therapy was most effective during the first two weeks after injury [80]. Later, by using bilateral closed femoral shaft fracture rat model, Wang *et al*. [81] and Yang *et al*. [82] demonstrated increased callus size after 7 daily 15-minute exposures to LIPUS treatment, compared with the contralateral controls. Our recent studies on LIPUS effects in rat closed fracture healing substantiated these early works and demonstrated the promoted early callus formation [17], [35], [38], [74].

LIPUS was also found to promote callus mineralization and remodeling in rats. As described earlier, from the radiographic analysis, the callus size of all the groups decreased rapidly, after reaching the peak value at week 2. It represented the start of the remodeling phase, overlapping significantly with the reparative phase, characterized by the slow modeling and remodeling of the fracture callus from woven to mature lamellar bone, and ultimately, the restoration of the bone to normal or near normal morphology and mechanical strength [83]. Among all the groups, the callus size in UG reduced more rapidly from week 2 to week 4 post-fracture, which suggested an accelerated remodeling process. The radiographic findings were supported by the results from µCT, which reflected the microstructure and mineralization of the callus. At week 4 post-fracture, we found BVh/TV and BMD in UG was significantly increased than in CG. Both BVh/TV and BMD are important tools for reflecting the degree of mineralization in callus. BMD of the callus has been used for the quantitative evaluation of the mechanical properties of healing bone [84]–[86]. The present results suggested LIPUS might accelerate the maturation process of callus. We also observed the apparent trend in the reduction of TV, BVl and BVl/TV in the callus of UG at week 4, as compared to in CG, although the differences were not statistically significant. These parameters reflected the reduced callus size and unmineralized tissue in the callus of UG, which was consistent with the radiographic analysis. The mechanical testing results further demonstrated the improved mechanical properties of callus under LIPUS treatment, as indicated by the higher UL and increased stiffness in UG at week 4. These findings were substantiated by the histological analysis, which showed the enhanced endochondral ossification in UG, in which fracture gap was better bridged at week 4 and filled with woven bone, whereas chondroid tissues were still present in the fracture area in CG.

Disrupting SDF1/CXCR4 signaling pathway by daily administration of AMD3100 on the rats receiving LIPUS treatment resulted in the significantly reduced GFP-positive cells in the fracture area, reduced callus size and less cartilage volume during fracture healing. These findings were supported by several previous studies. Toupadakis *et al*. examined the effect of AMD3100 on bone repair by using a murine fracture model. They found that the administration of AMD3100 led to a significantly reduced hyaline cartilage volume, callus volume and mineralized bone volume, associated with reduction of genes expression related to endochondral ossification [40]. Recently, Zhou found that in a traumatic brain injury/closed femoral fracture mice model, following AMD3100 treatment, the MSC migration was inhibited, and new bone formation was significantly reduced by 47% at the fracture sites in comparison with the controls treated with PBS [87]. However, when comparing to CG, UAG showed slightly better fracture healing, as shown by the early callus bridging (started from week 2), less chondroid tissues at fracture site and higher ultimate load. Taken together, these data suggest that SDF-1/CXCR4 signaling plays a very important role in fracture healing. Disrupting the SDF/CXCR4 pathway during LIPUS treatment could partially reduce, but not fully abolish LIPUS-induced fracture healing, indicating that other factors or signaling pathways might also be involved in this accelerated fracture repair process, such as increased blood circulation [88], upregulated osteogenic genes [89], and gap junctional cell-to-cell intercellular communication in rat MSCs [69].

The limitation of our study is that we used transplanted GFP-labeled allogenic MSCs to explore the migration activities of endogenous MSCs *in vivo*, which might not fully reflect the exact pattern of MSCs recruitment during facture healing. Further research may be necessary to understand the possible roles of endogenous MSCs in participating in tissue repair process.

## Conclusion

In conclusion, our study demonstrated that the application of LIPUS treatment could enhance SDF-1 signaling pathway, promote MSCs migration towards the fracture site, and accelerate fracture healing. This is the first evidence showing the micromechanical stimulation produced by LIPUS’s pressure waves can regulate MSCs migration through SDF-1/CXCR4 pathway in fracture healing. It provides novel insights into comprehensive mechanisms, through which LIPUS promotes fracture healing, and will potentially lead to the development of LIPUS enhanced MSC therapies for improving bone regeneration in a wide range of orthopaedic conditions.
